# Supplementary material for: Memristive FG–PVA Structures Fabricated with the Use of High Energy Xe Ion Irradiation
Source: Materials (Basel). 2022 Mar 11;15(6):2085. doi: 10.3390/ma15062085 (PMC8950800; doi:10.3390/ma15062085)
Supplement: Supplementary file 1 [file materials-15-02085-s001.zip › materials-1603917-supplementary.pdf]

Memristive FG/PVA structures fabricated with the use of high energy Xe ion irradiation

Artem I. Ivanov[1], Irina V. Antonova[1,2], Nadezhda A. Nebogatikova[1], Andrzej Olejniczak[3,4]

The polyvinyl alcohol used in the present study is short chains, and for this reason, PVA is a dielectric material. Fluorinated graphene before irradiation is also a dielectric material. The current-voltage characteristics for structures with an active layer created from PVA only or structures with FG layer are given in Fig. S1a,b. In the case of FG films, some resistance switchings are observed after irradiation with a maximum ON/OFF ratio of 1-2 orders of magnitude.

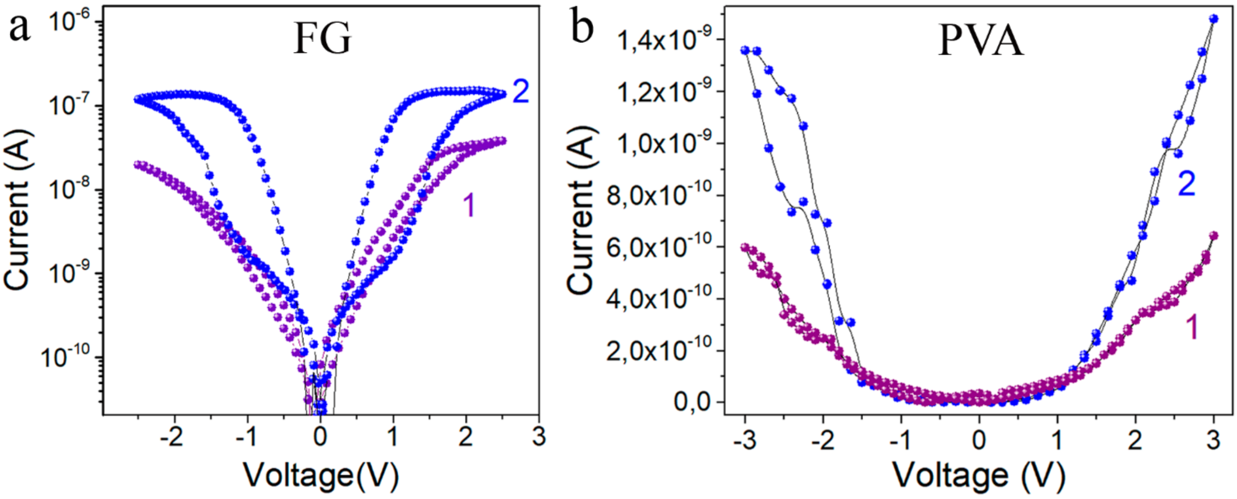

Figure S1. The current-voltage characteristics for structures with an active layer created from (a) FG layer only, (b) structures with PVA layer: curve 1 - before irradiation and curve 2 - after irradiation with 167 MeV ions and fluence of $3x10^{11}$ cm^{-2}. (c) The current-voltage characteristics for structures with active layers fabricated from the FG layer with GQDs.

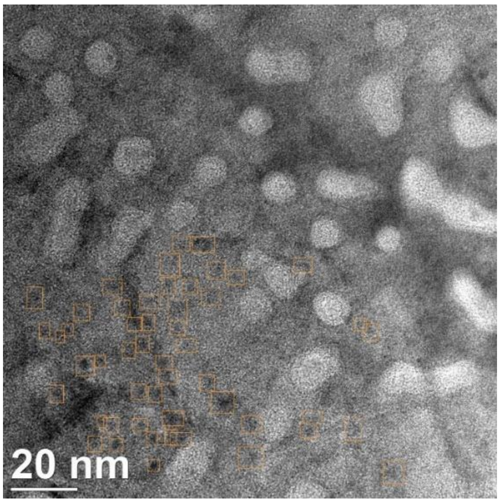

Figure S2. HREM images of GQDs in the FG films irradiated by 167-MeV Xe ions with a dose of $3x10^{11}$ cm^{-2}. Part of GQDs (small dark points) is marked by squares. The bright areas in images connect with surface relief of the thermally expanded FG particles.
